# The Internal Coherence of Breast Cancer Patients Is Associated with the Decision-Making for Chemotherapy and* Viscum album* L. Treatment

**DOI:** 10.1155/2018/1065271

**Published:** 2018-09-27

**Authors:** Shiao Li Oei, Anja Thronicke, Matthias Kröz, Cornelia Herbstreit, Friedemann Schad

**Affiliations:** ^1^Research Institute Havelhöhe, D-14089 Berlin, Germany; ^2^Hospital Havelhöhe, D-14089 Berlin, Germany; ^3^Charité–Universitätsmedizin Berlin, Corporate Member of Freie Universität Berlin, Humboldt-Universität zu Berlin, and Berlin Institute of Health, Germany; ^4^Institute for Integrative Medicine, University of Witten/Herdecke, D-58313 Witten/Herdecke, Germany

## Abstract

**Objective:**

In the present observational study, the influence of internal coherence on shared decision-making for chemotherapy (CTX) and* Viscum album *L. extracts (VA) treatment in breast cancer patients was evaluated.

**Methods:**

Breast cancer patients with a guideline-oriented advice from the tumor board for CTX were included in the study. At first diagnosis (T0) and 6 months later (T1), a questionnaire, the internal coherence scale (ICS), was administered and evaluated. Prior to analysis, patients were classified retrospectively depending on their treatment decision.

**Results:**

64 primary nonmetastasized breast cancer patients (median age 54.8 years, IQR: 46.3-65.3) were analyzed in this study. At T0, adjusted multivariable linear regression analyses revealed significant low ICS scores in patients rejecting CTX, especially in the ICS subscale “thermo coherence” (p = 0.006). The decision for add-on VA-therapy was associated with low scores for the ICS subscale “inner resilience coherence”, in particular low for the item “courage”. At T1, in the CTX+VA-group the thermo coherence increased significantly (p(d) < 0.01), while in contrast, in the CTX-only group the thermo coherence decreased significantly (p(d) = 0.02).

**Conclusion:**

Add-on VA-applications in CTX treatment support the thermo coherence of breast cancer patients, revealing a decision option to encourage patients to undergo CTX in combination with additional VA-treatments.

## 1. Introduction

Breast cancer is the most abundant malignant tumor in women worldwide [1]. Early detection methods and the establishment of improved treatment strategies reduced breast cancer mortality [2]. According to the German S3-guideline for early detection, diagnosis, therapy, and follow-up of breast cancer, adjuvant chemotherapy (CTX) is indicated for HER2-positive tumors, non-endocrine (or unclear) sensitive tumors, lymph node-positive disease, grade 3 tumors, luminal B tumors, and for patients aged < 35 years [3]. Decisions about adjuvant CTX in women with early stage breast cancer are often challenging [4]. Uncertainty about benefits versus evolving side-effects complicates decision-making. The factors that influence the treatment decisions are complex and involve issues regarding access to health care, concerns for cancer recurrence, and the impact of cancer therapies on health-related quality of life (HRQL). HRQL has gained growing importance in clinical oncology [5, 6]. Numerous questionnaires have been developed, to objectify and standardize HRQL analysis [7]; among them the mainly utilized is the European Organization for Research and Treatment of Cancer Quality of Life Questionnaire Core 30 (EORTC QLQ-C30) [8] and the Functional Assessment of Cancer Therapy (FACT) scale [9]. Beyond those captured dimensions of HRQL, other relevant aspects such as the sense of coherence [10, 11] and self-regulation [12] are more appropriate as prognostic outcomes in long-term observational studies of cancer patients [13]. The sense of coherence seems to have a positive impact on the HRQL [14]. As a prognostic clinical tool in oncology, a specific 10-item internal coherence scale (ICS) with self-reported questions capturing the individual skills of adaption, with good to very good reliability and sensitivity for treated cancer patients, was developed and validated [15]. During cancer treatment a comfort of well-being is often associated with good thermoregulation being a precondition on the subject of internal coherence [16, 17]. Therefore, in the ICS, items on thermo coherence are integrated. The structure of this ICS enables us to measure the total-ICS score and the subscales “inner resilience and coherence” and “thermo coherence” [15]. Moreover, this questionnaire especially has a good responsiveness during CTX and shows some sensitivity for VA-treatment [18].

Women with breast carcinoma appear to have a strong desire for involvement in making decisions regarding their treatment [4]. Patient's expectations and motivations for using complementary alternative medicine are often chosen to manage pain or other treatment-related side-effects [19]. The feeling of being active involved in treatment decisions, with an associated level of self-control, is an important motivation for patients to use complementary alternative medicine [20].* Viscum album *L. extracts (VA, mistletoe) are frequently used in integrative oncology to reduce treatment-related side-effects [21, 22]. VA-applications are usually well tolerated and had only few and mild side-effects [23–25]. Various reports support clinical findings that VA-therapy can improve and stabilize HRQL in cancer patients, especially in breast cancer during CTX [26–29]. In this noninterventional, observational study, the ICS was used to evaluate the effects of add-on VA-therapy on the internal coherence in breast cancer patients with the advice for CTX. Firstly, the ICS at first diagnosis was analyzed to compare the individual internal coherence of the patients in regard to their decision for CTX and/or add-on VA-treatment. Furthermore, the change on the ICS, in particular the thermo coherence score of the patients during therapy, 6 months after first diagnosis was followed. In addition, occurring tumor recurrences within 3 years after first diagnosis were evaluated for the study group.

## 2. Methods

### 2.1. Study Design

We conducted a prospective observational study for primary stage I-III breast cancer patients as reported previously [30, 31]. Patients who gave written consent to be registered in the Network Oncology (NO) registry [32], who were diagnosed between June 2012 and April 2017 at the certified Breast Cancer Centre (Gemeinschaftskrankenhaus Havelhöhe, Berlin, Germany), and who received a tumor board advice for CTX were included. Follow-up examinations were performed until December 2017 and only patients were included here from which completed ICS questionnaires at first diagnosis (T0) and 6 months after first diagnosis (T1) were available. Patients who received no advice for CTX were excluded. The NO registry has been approved by the ethics committee of the Medical Association Berlin (Eth-27/10).

### 2.2. Data Collection

The NO registry utilizes linked data from primary and secondary care in concert with cancer registry data and hospital episode statistics. Demographic data as well as information on diagnosis, histology, surgery, radiation, and previous treatment regimen were retrieved from the NO registry [32]. Effects were evaluated at first diagnosis (T0) and 6 months (T1) after first diagnosis. The occurrence of recurrences was monitored until December 2017.

### 2.3. Classification of Groups

Classification of groups was performed retrospectively, according to the decision of the patients to follow the advice for CTX and/or to make use of receiving add-on VA-therapy. Patients who received CTX only were allocated to the CTX- group (n = 25). Patients who received CTX and add-on VA were allocated to the CTX+VA-group (n = 31). Patients, rejecting CTX but receiving add-on VA, were allocated to the VA- group (n = 8). Generally, CTX- and VA-therapy, when applied, started within six months after first diagnosis.

### 2.4. Endpoints

Patient-reported outcomes were evaluated by analyzing the ICS in all groups. The ICS is a short, highly reliable, and valid ten-item self-reported questionnaire based on a 5-point Likert scale (range: 10 (low ICS) – 50 (high ICS)). The ICS contains two subscales, one with eight items (Inner Coherence and Resilience) and a second subscale (thermo coherence) with two items. The internal consistency (Cronbach-alpha) is with r = 0.91 robust as well as test-retest-reliability (after 4-8 weeks) is classified as good (r = 0.80) with sufficient validity [15]. The ICS was evaluated for T0 and T1. For the evaluation of the occurrence of recurrences the collected data of the tumor board case conferences were analyzed.

### 2.5. Statistical Analysis

Continuous variables were described as median with interquartile range (IQR); categorical variables were summarized as frequencies and percentages. p-values < 0.05 were considered to be significant. All statistical analyses were performed using the software R (R Version 3.1.2 (2014)) [33]. For Pearson's chi-square calculation, the basic R-package was used, and for Cohen's d analyses in addition the “compute.es” package was incorporated. For all groups, baseline characteristics and treatment regimens were compared by the calculation of Pearson's chi-squares and p-values. For further ICS-quantifications adjusted multivariable linear regression analyses were performed. Statistical models were applied for all single questions, sub- and total-scales. Predicting variables were age (in years), body mass index (BMI, continuous variable), UICC stages (categorical I, II, III), hormonal status (pre-/peri-/postmenopausal), estrogen receptor positive (yes/no), progesterone receptor positive (yes/no), and HER2 positive (yes/no).

## 3. Results

### 3.1. Patient Characteristics

**Figure 1:**
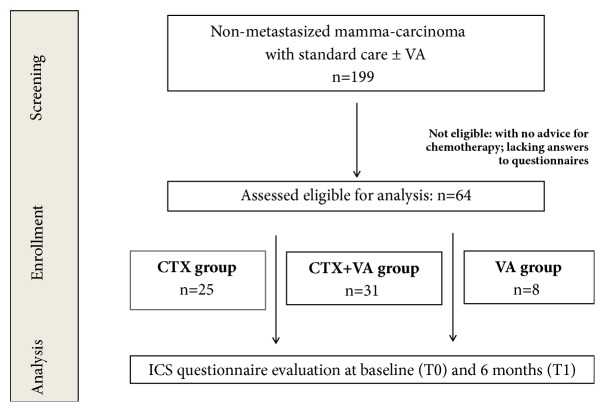
**Flow chart of the study population.** Classification of groups was performed according to the treatment decision of the patients. CTX-group: patients treated only with guideline-oriented CTX; CTX+VA-group: patients treated with a combination CTX and add-on VA-extracts; VA-group: patients rejecting CTX but receiving add-on VA-extracts.

Complete data were collected for 64 eligible women with nonmetastasized breast cancer, which had, as a result of a multidisciplinary tumor board case conference, the advice for CTX. Medium age was 54.8 years, IQR was 46.3-65.3, and the hormonal status of 51.6% was postmenopausal; for details see Table 1. The first ICS (T0) were administered before onset of systemic anticancer therapy, usually after cancer-related surgery. The patients were assigned to the groups according to their decision, to follow the recommended CTX and/or to receive add-on VA-extracts. The patients of the CTX- (n = 25) and CTX+VA- (n = 31) groups received CTX without or with additional VA-therapy. The 8 patients of the VA-group rejected CTX but received VA-therapy (see also study flow chart, Figure 1). Table 1 shows the main characteristics of patients of the groups at baseline. Significant differences between the groups were obtained for the patients, especially regarding their hormonal status (p = 0.008). In the CTX-group the majority of patients were premenopausal while in the other groups most patients were postmenopausal. According to advised oncologic therapy, generally, anticancer treatments started within 6 months after first diagnosis and were administered to the patients for several years. VA-extracts of different producers, when applied, were given mostly as subcutaneous injections (for details see Table 2).

### 3.2. Decision-Making and ICS at First Diagnosis

**Figure 2:**
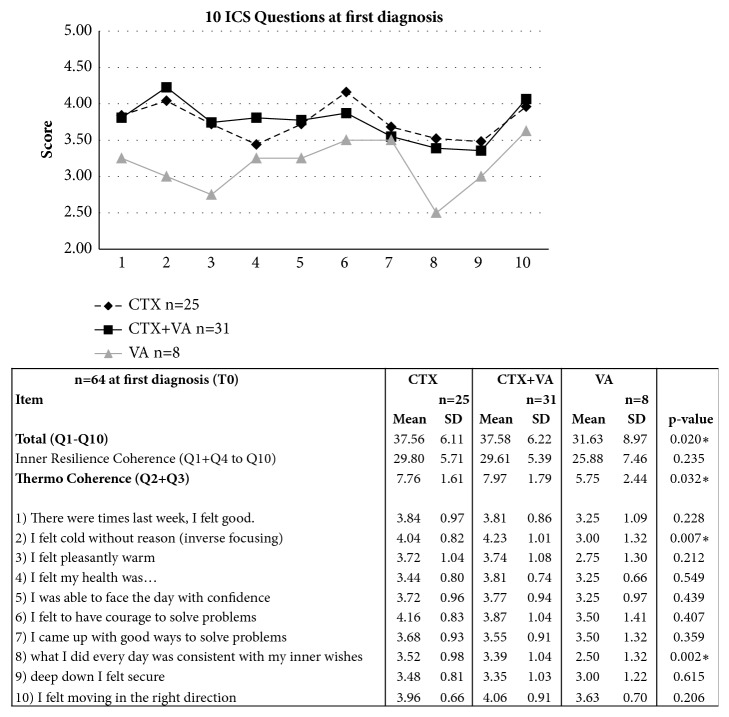
**ICS questionnaire of breast cancer patients.** The 10 items of the ICS questionnaire with answer possibilities 1-5 (1 = low ICS, 5 = high ICS). In the upper diagram the profile of answer score of all 10 items for all groups is shown for the values at first diagnosis. Subscales, mean values, standard deviations, and p-values were calculated.

The ICS was administered to all patients at first diagnosis before final decision for anticancer interventions and onset of oncological care. In Figure 2, the 10 items of the ICS are listed, and the mean values of all 10 queries were determined for the CTX, CTX+VA, and VA-groups, respectively.

In Figure 2 on the upper diagram, the profile of answer score for the three groups is shown for the mean values at T0. The total-ICS and subscores were calculated. At T0, before the onset of oncological care, strong and significant differences were obtained between the groups. Chi-square analyses and p-values determinations between the groups revealed significant differences for the total-ICS, the thermo coherence, and some single question scores (table in Figure 2). Demographic variables were considered to adjust these differences (Table 3). For the total-ICS the regression analysis revealed a very large and significant low total-ICS for the VA-group (-7.521, p = 0.005). Also the total-ICS of the CTX+VA-group in reference to the CTX-group was lower, although not significant (-2.691, p = 0.152). Notably, a rather low total-ICS was significantly associated with the perimenopausal status (-7.638, p = 0.015) and a higher total-ICS (although not significantly) with the postmenopausal status (+4.705, p = 0.069). Moreover, the total-ICS was associated with a lowered BMI (-0.384, p = 0.019). In further multivariable regression analyses in Table 3, age, BMI, and hormonal status were considered and instead of the group, the decisions for CTX or VA-therapy, respectively, were included as variables. There was a negative association between the inner resilience and coherence subscale, and the decision for VA-therapy was nearly significant (-3.076, p = 0.056). Similar as for the total-ICS, the inner resilience and coherence were significantly associated with a lowered BMI and with a perimenopausal status. Furthermore, an increased thermo coherence score was significantly associated with the decision for CTX. With regard to the hormonal status, a postmenopausal status was significantly associated with a higher thermo coherence score (+1.507, p = 0.037) while no considerable thermo coherence differences were obtained between pre- and perimenopausal patients. Among the means of the scores for single questions, significant differences between the groups were obtained for question 2 (feeling cold; p = 0.007) and question 8 (what I did every day was consistent with my inner wishes; p = 0.002) (see Figure 2). In Table 4 multivariable regression analyses for scores of single questions, considering age, BMI, hormonal status, and decision for CTX or VA-therapy, respectively, are shown. High scores for question 2 (this means, rarer feeling cold without reason) were significantly associated with the decision for CTX (+0.903 with p = 0.031) and also for the postmenopausal status (+1.132, p = 0.009). Furthermore, the decision for CTX was associated with a significant high score for question 8 (consistency with my inner wishes) (+1.108, p = 0.013). In addition, further striking differences were obtained. In particular, a low score for question 6 (this means, rarer having courage to solve problems) seemed to be associated with a perimenopausal status (-1.115 with p = 0.036) and also for the decision for VA-therapy (-0.623, 0.049).

### 3.3. Thermo Coherence

**Figure 3:**
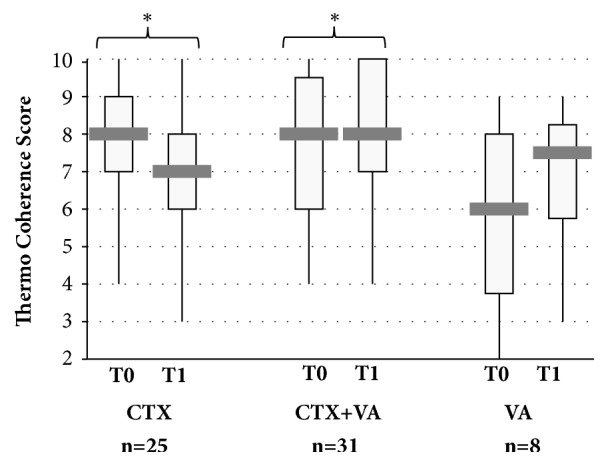
**Thermo coherence during therapy of breast cancer patients. **Boxplots for the thermo coherence scores for the CTX-, CTX+VA, and VA-group at first diagnosis (T0) and 6 months later (T1). ^*∗*^Calculation of Cohen's d revealed for the CTX* d*[95% CI] = 0.70 [0.09, 1.31], p(d) = 0.02^*∗*^ and for the CTX+VA-group* d* [95% CI] = 0.96 [0.39, 1.53], p(d) < 0.01.

In Figure 3 boxplots for the thermo coherence for all groups at first diagnosis (T0) and 6 months of follow-up (T1) are shown. For the CTX-group the thermo coherence score decreased significantly during chemotherapy. Pearson's chi-squared test and Cohen's d analyses revealed a significant medium effect size, *d* [95% CI] = 0.70 [0.09, 1.31], p(d) = 0.02 for this decrease. In contrast, for the other groups increased thermo coherence scores were detected during VA-therapy (Figure 3). For the CTX+VA-group a significant effect was obtained, *d* [95% CI] = 0.96 [0.39, 1.53], p(d) < 0.01 for this increase. Similarly, an increase of the thermo coherence score was obtained for the VA-group; however, due to the small sample size these analyses were not yet statistically significant (*d* [95%CI] = 0.99 [-0.25, 2.24], p(d) = 0.11).

### 3.4. Occurrence of Recurrences

**Figure 4:**
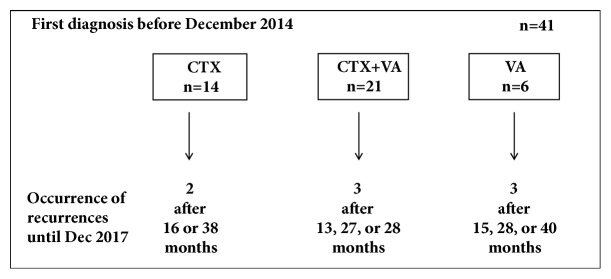
**Three-year follow-up of tumor recurrences. **The occurrence of tumor recurrences was evaluated for the patients (n = 41) with first diagnosis before December 2014.

The first diagnoses of the entire study population (n = 64) were carried out between March 2012 and February 2017. For a three-year follow-up evaluation of the occurrence of tumor recurrences only the patients (n = 41) with first diagnosis before December 2014 were taken into consideration (14 CTX, 21 CTX+VA, and 6 VA). Until December 2017 in 8 from 41 cases (19.5%), recurrences were detected within 13- 40 months after their first diagnosis (Figure 4). The rates of occurrence of recurrences in the CTX and in the CTX+VA-groups were comparable, about 14% (2 in the CTX-group and 3 in the CTX+VA-group), while in 3 patients of the CTX rejecting group (VA-group) recurrences were detected (50%). Of the 8 patients with recurrences, 3 of them rejected CTX and radiation, 3 have had UICC-Class I, 4 have had UICC-Class II, and 1 has had UICC-Class III tumors, and 6 of them were HER2 positive. 3 patients were premenopausal, 4 were postmenopausal, and 1 was perimenopausal.

## 4. Discussion

The results of the present study reveal that the individual inner coherence and hormonal status of breast cancer patients seem to be crucial for decision finding for cancer therapy. Strikingly, low scores for consistency with inner wishes and thermo coherence were associated with the decision against CTX. On the other side, a low score of the inner resilience coherence, especially rarer having courage to solve problems, is significantly associated with the decision for an additional VA-therapy. Furthermore, for patients treated additionally with VA-extracts, their thermo coherence scores increased significantly 6 months after first diagnosis.

At time of first diagnosis, before patients decided for the respective oncological treatment, common features as well as significant differences in the individual ICS-profiles were observed, although these ICS-queries were not integrated in the decision-finding process. As being reported previously, the ICS is associated with gender and age [15]. In line with this finding, we observed significant associations between the hormonal status and the ICS of the patients in the present study. For the perimenopausal patients the scores of the inner resilience and coherence were significantly low, while the thermo coherence of postmenopausal patients was significantly high. A thermal dysregulation has been seen particularly in premenopausal breast cancer patients and can be related to explicitly affect the HRQL [34]. Notably, CTX increases fatigue levels [35] and has been linked to “feeling cold” [17]. Moreover, the symptoms, such as “feeling cold” or hot flushes and congestive sweating, have been discussed as a constitutional pattern in premenopausal breast cancer patients [34]. In line with these expectations, a significant decline of the thermo coherence score in the present study was found 6 months after onset of CTX but only for the CTX-group (Figure 3). In contrast, the thermo coherence scores of the other groups which were treated with VA-extracts increased, suggesting that VA-applications might support thermoregulation in breast cancer patients and this may in part alleviate thermal discomfort. Reduced internal coherence scale values in association with loss of autonomic, rest/activity, and orthostatic-circulatory regulation in breast cancer patients were reported in another case-control study [36].

The rates of occurring recurrences in the CTX- related to the CTX+VA-group were comparable and at present date, in 50% of patients rejecting CTX towards just 14% of patients receiving CTX, developing recurrences were detected. Even if these preliminary analyses did not yield significant group differences between the recurrence rates, the necessity of undergoing CTX should not be called into question. Thus, there is an urgent need to encourage patients rejecting CTX and to offer them an efficient treatment regimen by reducing adverse effects to a bearable extent.

CTX is known to negatively affect HRQL [35]. Clinician's recommendations play a significant role in either accepting or declining CTX. For instance, Harder et al. [37] reported that older women with early breast cancer preferred to be involved in clinical decision-making. Medical oncologists should be able to apply decision aid modalities in a personalized manner to give all needed information to their patients, thereby ensuring a deliberate shared decision-making process [4] and facilitating acceptance of a need for CTX. Thus, any treatment implemented should be in harmony with the patient's preferences [38]. Currently, there is no guideline for the treatment of patients who reject CTX. In the certified Breast Cancer Center of the present study, informed decision-making is performed according to the German S3-guideline for early detection, diagnosis, therapy, and follow-up of breast cancer [3] but it also takes into account the individual life situation and needs of the patients.

## 5. Conclusions

In the present analysis it is shown that patients with a low internal coherence, especially thermo coherence, tend to reject immediate CTX. On the other side, it is demonstrated that VA-applications led to a better thermoregulation. Thus add-on VA might present a decision option, to encourage patients to undergo CTX, at least at a later time, to ensure prevention of progress of tumor development.

## Figures and Tables

**Table 1:** Baseline characteristics of primary breast cancer patients (n = 64) at day of first diagnosis.

	**Total**	**CTX**	**CTX+VA**	**VA**	**p-value**
**Number of patients, n (**%**)**	64 (100)	25 (100)	31 (100)	8 (100)	
Age, years, median (IQR)	54.8 (46.3-65.3)	50.5 (44.0-52.0)	56.9 (49.9-65.3)	59.9 (51.0-76.2)	0.155
BMI, median (IQR)	25.9 (22.7-28.0)	26.3 (23.0-28.8)	25.3 (22.2-28.2)	26.8 (23.0-27.4)	0.231

**UICC stages, n (**%**)**					
I	24 (37.5)	9 (36.0)	13 (41.9)	2 (25.0)	
II	26 (40.6)	10 (40.0)	12 (38.7)	4 (50.0)	0.926
III	14 (21.9)	6 (24.0)	6 (19.4)	2 (25.0)	

**Hormonal status, n (**%**)**					
Pre-menopausal	26 (40.6)	15 (60.0)	8 (25.8)	3 (37.5)	
Peri-menopausal	5 (7.8)	4 (16.0)	1 (3.2)	0 (0)	0.008^*∗*^
Post-menopausal	33 (51.6)	6 (24.0)	22 (71.0)	5 (62.5)	

Estrogen receptor positive	41 (64.1)	15 (60.0)	19 (61.3)	7 (87.5)	0.334
Progesteron receptor positive	40 (62.5)	16 (64.0)	19 (61.3)	5 (62.5)	0.979
HER 2 positive	23 (35.9)	7 (28.0)	14 (45.2)	2 (25.5)	0.325

**Interventions, n (**%**)**					
CTX, n (%)	56 (87.5)	25 (100)	31 (100)	0 (0)	<0.001^*∗∗*^
VA therapy, n (%)	39 (60.9)	0 (0)	31 (100)	8 (100)	<0.001^*∗∗*^
Radiation, n (%)	44 (68.8)	19 (76)	22 (88.0)	3 (37.5)	0.115

TNM staging according to the Union for International Cancer Control (UICC). n = number of patients and portion (%). CTX = chemotherapy; VA = *Viscum album* L. therapy; BMI: body mass index. The p-values of Pearson's chi-square analyses of the three groups were determined using R-statistics.

**Table 2:** Characteristics of VA-therapy.

	**Patients, n**
**Total number of VA patients, n (**%**) **	39 (100)

**Preparations, n (**%**)**	
Abnobaviscum	24 (61.5)
Iscador	8 (20.5)
Helixor	14 (35.9)
Iscucin	4 (10.3)

**Application, n (**%**)**	
subcutaneous	33 (84.6)
intravenous	18 (46.2)

Characteristics of VA-therapy applied additionally to standard of care (n = 39). Numbers in columns do not necessarily add to 39 as patients may have received various VA combinations of preparations and applications, respectively.

**Table 3:** Association factors for ICS and subscores.

n = 64	**Total ICS**			**Inner resilience coherence**			**Thermo coherence**		
	**estimate**	**SE**	**p-value**	**estimate**	**SE**	**p-value**	**estimate**	**SE**	**p-value**
**demographic variables**									
Age	-0.135	0.092	0.147	-0.099	0.081	0.226	-0.053	0.027	0.055
BMI	**-0.384**	**0.159**	**0.019** ^**∗**^	**-0.362**	**0.142**	**0.014** ^**∗**^	-0.035	0.047	0.458

**Hormonal status**									
Pre-menopausal	reference			reference			reference		
Peri-menopausal	**-7.638**	**3.037**	**0.015** ^**∗**^	**-6.930**	**2.716**	**0.013** ^**∗**^	-0.357	0.903	0.694
Post-menopausal	4.705	2.536	0.069	3.604	2.237	0.112	**1.507**	**0.707**	**0.037** ^**∗**^

**Group **					**Decision**			**Decision**	
**Reference**		**CTX**			**no VA-**			**no CTX-**	
**CTX+VA**	-2.691	1.852	0.152	**-3.076**	**1.580**	**0.056**	**2.000**	**0.697**	**0.006** ^**∗∗**^
** VA**	**-7.521**	**2.574**	**0.005** ^**∗∗**^		**VA-therapy**			**CTX**	

Multivariable linear regression analyses using R-statistics were performed for decision making and the total-ICS, the inner resilience coherence, and thermo coherence at T0. CTX group: patients treated only with guideline-oriented CTX; CTX+VA group: patients treated with a combination CTX and add-on VA-extracts; VA-group: patients rejecting CTX but receiving add-on VA-extracts.

**Table 4:** Association factors for ICS single scores.

**n = 64 **	**Question 2:**			**Question 6:**			**Question 8: Consistency **		
	**I felt cold without reason**			**Courage to solve problems**			**with my inner wishes**		
	**estimate**	**SE**	**p-value**	**estimate**	**SE**	**p-value**	**estimate**	**SE**	**p-value**
**demographic variables**									
Age	**-0.042**	**0.015**	**0.008** ^**∗∗**^	-0.027	0.016	0.09	0.005	0.016	0.762
BMI	-0.014	0.026	0.591	-0.033	0.026	0.225	-0.03	0.027	0.277

**Hormonal status**									
Pre-menopausal	reference			reference			reference		
Peri-menopausal	-0.021	0.508	0.968	**-1.115**	**0.519**	**0.036** ^**∗**^	**-1.300**	**0.536**	**0.019** ^**∗**^
Post-menopausal	**1.132**	**0.42**	**0.009** ^**∗∗**^	0.645	0.43	0.139	-0.072	0.444	0.872

***Receptor negative***	reference			reference			reference		
Estrogen positive	-0.204	0.377	0.59	-0.139	0.385	0.72	1.068	0.4	0.010^**∗**^
Prog positive	0.278	0.361	0.445	0.002	0.369	0.996	-0.697	0.381	0.073
HER2 positive	0.163	0.275	0.555	0.171	0.281	0.547	0.338	0.291	0.249

**Decision **									
**CTX**^**1**^	**0.903**	**0.408**	**0.031** ^**∗**^	0.153	0.418	0.715	**1.108**	**0.431**	**0.013** ^**∗**^
**VA**^**2**^	-0.114	0.302	0.707	**-0.623**	**0.309**	**0.049** ^**∗**^	-0.417	0.319	0.197

Multivariable regression analyses for the single questions 2 (I felt cold without reason), 6 (courage to solve problems), and 8 (consistent with my inner wishes). References for treatment decisions were ^1^no CTX or ^2^no VA-therapy.

## Data Availability

The datasets used and/or analyzed during the current study have been kept confidential and are not available publicly.
